# Australian Real‐World Effectiveness and Safety of Ustekinumab for the Treatment of Crohn's Disease: Results of the AURORA Study, Including the ANZIBD Consortium

**DOI:** 10.1002/jgh3.70322

**Published:** 2025-12-09

**Authors:** Yoon‐Kyo An, Niamh Lindsay, Natalie Allan, Emi Khoo, Richard Fernandes, Robert Gilmore, Anna Amiss, Hai Pham, Soong‐Yuan Ooi, Lena Thin, Daniel Lightowler, Susan J. Connor, Astrid Williams, Peter De Cruz, Christopher F. D. Li Wai Suen, Viraj Kariyawasam, Nikola Mitrev, Simon Ghaly, Jane M. Andrews, Britt Christensen, Miles P. Sparrow, Lauren S. White, Robert Bryant, Nik S. Ding, Rupert W. Leong, Daniel R. Van Langenberg, Hansjoerg Seltenreich, Kavitha Subramaniam, Graham Radford‐Smith, Jakob Begun

**Affiliations:** ^1^ Mater Hospital Brisbane Brisbane Queensland Australia; ^2^ Mater Research Institute the University of Queensland South Brisbane Queensland Australia; ^3^ Royal Brisbane and Women's Hospital Herston Queensland Australia; ^4^ Fiona Stanley Hospital Murdoch Western Australia Australia; ^5^ The University of Western Australia Perth Western Australia Australia; ^6^ Liverpool Hospital Liverpool New South Wales Australia; ^7^ University of New South Wales Sydney New South Wales Australia; ^8^ Austin Hospital Heidelberg Victoria Australia; ^9^ The University of Melbourne Melbourne Victoria Australia; ^10^ Blacktown & Mount Druitt Hospital Blacktown New South Wales Australia; ^11^ St Vincent's Hospital Sydney Sydney New South Wales Australia; ^12^ Royal Adelaide Hospital Adelaide South Australia Australia; ^13^ The University of Adelaide Adelaide South Australia Australia; ^14^ The Royal Melbourne Hospital Parkville Victoria Australia; ^15^ Alfred Health and Monash University Melbourne Victoria Australia; ^16^ Sunshine Coast University Hospital Sunshine Coast Queensland Australia; ^17^ The Queen Elizabeth Hospital Woodville South South Australia Australia; ^18^ St Vincent's Hospital Melbourne Fitzroy Victoria Australia; ^19^ Concord Repatriation General Hospital Concord New South Wales Australia; ^20^ University of Sydney Sydney New South Wales Australia; ^21^ Eastern Health Box Hill Victoria Australia; ^22^ Coastal Digestive Health Maroochydore Queensland Australia; ^23^ Canberra Hospital Canberra Australian Capital Territory Australia; ^24^ Australian National University Canberra Australia; ^25^ QIMR Berghofer Medical Research Institute Herston Queensland Australia; ^26^ Faculty of Medicine University of Queensland Brisbane Queensland Australia

**Keywords:** Australia, Crohn's disease, real‐world, Ustekinumab

## Abstract

**Background:**

Ustekinumab is an effective therapy for the management of Crohn's disease. Australia is unique, as ustekinumab can be prescribed as first‐line biologic therapy, and there is high concomitant immunomodulator use.

**Aim:**

To evaluate the real‐world efficacy and safety of ustekinumab in moderate to severe Crohn's disease.

**Methods:**

A multicentre prospective cohort study was conducted at 19 Australian centres between September 2019 and April 2022. Clinical assessments were performed at baseline, 3, 9 and 15 months. Logistic regression analyses were performed to identify predictors of clinical response and remission.

**Results:**

197 patients (male 45.2%) were included: 58.9% were biologic‐naïve and 50.0% were on concomitant immunomodulators. Clinical response rates were 75.4%, 75.5% and 72.3% at 3, 9 and 15 months, respectively with corresponding clinical remission rates of 45.8%, 51.6% and 55.5%. Clinical response and remission rates at 3 and 9 months were significantly higher in bio‐naïve patients compared with biologic‐exposed (*p* < 0.01); but no significant differences were seen with concomitant immunomodulator use. Dose escalation was required in 31.5% of patients. Ustekinumab was discontinued in 12.7% of patients. The cumulative probability of maintaining ustekinumab treatment at 15 months was 84.4%. Despite 161 adverse events reported, including 41 hospitalizations, only eight patients required treatment discontinuation due to adverse events.

**Conclusions:**

This real‐world study on the use of ustekinumab in Crohn's disease showed that short‐term clinical response and remission rates are higher in bio‐naïve compared with bio‐exposed patients, with a high persistence rate at 15 months. The addition of an immunomodulator did not significantly impact outcomes. Ustekinumab was found to be safe in most patients.

## 1 Introduction

Inflammatory bowel disease (IBD) is a chronic inflammatory disorder of the gastrointestinal tract divided into two main conditions, ulcerative colitis and Crohn's disease (CD). In Australia, CD affects more than 40,000 individuals, with an annual incidence rate of 29.3 per 100 000, one of the highest globally [1]. The inflammatory process in CD primarily affects the gastrointestinal tract and is often most severe in the terminal ileum, with other affected sites including the upper gastrointestinal tract, colon, and perineum [2]. The treatment landscape for CD has evolved over the years, with the emergence of targeted biologic therapies offering a new possibility for improved patient outcomes [3]. Individuals within the Australian health care system with active CD are typically required to have corticosteroid (in Australia this refers to oral prednisolone) refractory disease together with either intolerance or disease that is refractory to immunomodulators (thiopurines or methotrexate) prior to accessing anti‐tumor necrosis factor (TNF) alpha antagonists, vedolizumab, or ustekinumab (UST), via the Australian pharmaceutical benefits scheme (PBS).

UST is a human monoclonal antibody targeting the interleukin (IL)‐12 and IL‐23 pathway by binding to their shared p40 subunit. It has been available for Australian patients with moderate to severe luminal CD through the PBS scheme since September 2017. While UST presents a compelling therapeutic option [3, 4, 5, 6, 7], gaps in understanding its role in clinical practice persist, largely due to a lack of comparative effectiveness studies and limitations in existing real‐world studies. One multicentre trial compared randomized, controlled, head‐to‐head outcomes of treatment with UST or Adalimumab (ADA) for bio‐naïve patients with moderately to severely active CD [8]. Sands et al. showed high rates of clinical remission with both UST and ADA after 52 weeks with no significant difference between medications (65% vs. 61%, 95% CI −6 to 14, *p* = 0.42). In comparison, the current real‐world studies, often retrospective and derived from single‐centre settings with small sample sizes and short durations of follow‐up, fail to provide comprehensive insights into the effectiveness of UST and patient factors associated with response. Additionally, most patients in these studies have experienced prior biologic failures, in part because of prescribing restrictions in certain regions that reserve UST as a second‐line biologic choice. Thus, there is a need for more extensive real‐world data from broad and diverse patient populations to address these limitations and better determine the effectiveness of UST.

The Australian context presents a unique opportunity for real‐world observational studies because UST can be prescribed as a first‐line advanced therapy, allowing inclusion of a higher proportion of patients who are biological naïve. Moreover, the strict prescribing regulations established by the Australian PBS lead to high utilization of concomitant immunomodulators, allowing evaluation of response to both combination and monotherapy with UST in CD. To bridge the existing gaps in real‐world studies and offer more robust evidence, we undertook a large‐scale Australian multicentre prospective cohort study. This study aimed to evaluate the efficacy and safety of UST in moderate to severe CD patients and to identify clinical and biomarker predictors of UST response and remission.

## 2 Methods

### 2.1 Study Design

We conducted a nationwide, multicentre, prospective cohort study to investigate the efficacy and safety of UST therapy for moderate‐to‐severe CD refractory to immunomodulators and corticosteroids. The study design was aligned with the criteria specified for biologic therapy reimbursement under the Australian PBS. In Australia, prescribing biologic therapy for CD requires a physician to demonstrate persistent active disease (Crohn's Disease Activity Index [CDAI] ≥ 300 or CDAI ≥ 220 with extensive small bowel disease) despite prior optimisation with corticosteroids and either the use of immunomodulators or intolerance to them. Following the initiation of biologic therapy, physicians must demonstrate an adequate and sustained clinical response (CDAI decrease to < 150, or improvement of intestinal inflammation) within 3 months, and continued response every 6 months for continuation of maintenance therapy.

Patients were recruited from 19 Australian IBD centres, including 12 sites within the Australia and New Zealand IBD Consortium (ANZIBDC), between September 2019 and April 2022. The study included men and women aged between 18 and 80 at the time of informed consent with moderately‐to‐severely active CD of at least 3 months' duration since diagnosis, and eligible for UST treatment through the PBS.

There were four study visits, including the baseline visit, over a follow‐up period of 15 months. All study visits adhered to standard care to comply with PBS assessment periods. Baseline clinical evaluations were conducted at the first study visit (SV1, the day UST treatment commenced), and the post‐induction assessment occurred at approximately 3 months (SV2, between weeks 6–18). Subsequent evaluation took place every 6 months at approximately 9 months (SV3, between weeks 28–44) and 15 months (SV4, between weeks 52–68). During each visit, patients received standard clinical care by their IBD treatment team, involving clinical assessments and standard local laboratory pathology tests, including blood and stool analysis. Serum samples for UST levels were collected post‐induction at SV2 and were analyzed using an enzyme‐linked immunosorbent assay (ELISA; Promonitor‐UTK, Grifols, Derio, Spain). A subset of patients underwent endoscopy and intestinal imaging (CT, MRI or intestinal ultrasound) at baseline and throughout the study period, with results documented at each visit. All study data were recorded in purpose‐designed paper case report forms (CRF) and entered into REDCap electronic data capture tools hosted at the University of Queensland [9, 10]. REDCap (Research Electronic Data Capture) is a secure, web‐based software accessible remotely by authorized personnel. Data entry was monitored by a central data manager, ensuring accurate and timely entry, with the REDCap data validated against the CRF.

As part of standard care, patients received a single weight‐based UST intravenous (IV) induction dose at week 0 (260 mg for weight ≤ 55 kg; 390 mg for weight > 55 kg and ≤ 85 kg; 520 mg for weight > 85 kg). Subsequently at week 8, and every 8 weeks thereafter, patients received standard UST 90 mg subcutaneous (SC) maintenance doses until the end of the study period. Patients with an inadequate response underwent UST dose escalation at the discretion of the treating clinician, through a compassionate dosing program.

### 2.2 Outcomes and Definitions

The primary objective of the study was to evaluate the effectiveness of UST in achieving clinical response and remission at 3, 9 and 15 months after initiation of therapy. Following the STRIDE‐II guidelines [11], clinical response was defined as a decrease of at least 50% in patient‐reported outcome 2 (PRO2) which includes abdominal pain and stool frequency. Clinical remission was defined as a PRO2 score where abdominal pain ≤ 1 and stool frequency ≤ 3.

Secondary objectives included the determination of the proportion of patients achieving various endpoints: (i) corticosteroid‐free response and remission (as per definitions of clinical response and remission, and corticosteroid withdrawal in patients who were receiving corticosteroids at baseline) at 3, 9 and 15 months; (ii) biochemical response (C‐reactive protein [CRP] or fecal calprotectin [FC] reduction of at least 50%) and biochemical remission (CRP or FC level normalization [≤ 5 mg/L and < 250 μg/g, respectively]) at 3, 9 and 15 months; (iii) treatment persistence at 3, 9 and 15 months; (iv) frequency of dose escalation; (v) post‐induction UST drug concentration on clinical response and remission at 3 and 15 months; and (vi) adverse event and serious adverse event at 15 months.

### 2.3 Statistical Analysis

All analyses were performed using R version 4.2.2 (R Foundation for Statistical Computing, Vienna, Austria). Baseline patient and clinical characteristics were summarized using standard descriptive statistics, specifically median (quartile 1, quartile 3) for continuous variables and frequency (%) for categorical variables. Differences in baseline characteristics between groups were compared using Pearson's Chi‐squared tests or Fisher's exact tests (for categorical variables) and Wilcoxon rank‐sum tests (for continuous variables). The significance level was set at *p* < 0.05, and no adjustments for multiple testing were made.

For clinical response and remission, data were reported using hybrid approaches, including (i) as observed, (ii) non‐responder imputation (NRI) and last observation carried forward (LOCF) analysis. In NRI analysis, missing data were imputed as treatment failure, while in LOCF analysis, the assumption was made that the response remained constant at the last observed value. Kaplan–Meier curves were generated to illustrate UST discontinuation.

Univariable and multivariable logistic regression models were employed to assess associations between baseline clinical and biochemical factors and clinical response and remission. All variables with *p* values < 0.20 on univariate analysis were included in the multivariable model, and stepwise backward regression was conducted to further exclude any variable with *p* value > 0.50 in the multivariable model. For consistency, we favored the inclusion of history of extraintestinal manifestation (EIMs) in the multivariable model over arthralgia, as EIM was primarily represented by arthralgia.

## 3 Results

### 3.1 Baseline Patient Characteristics

**FIGURE 1:**
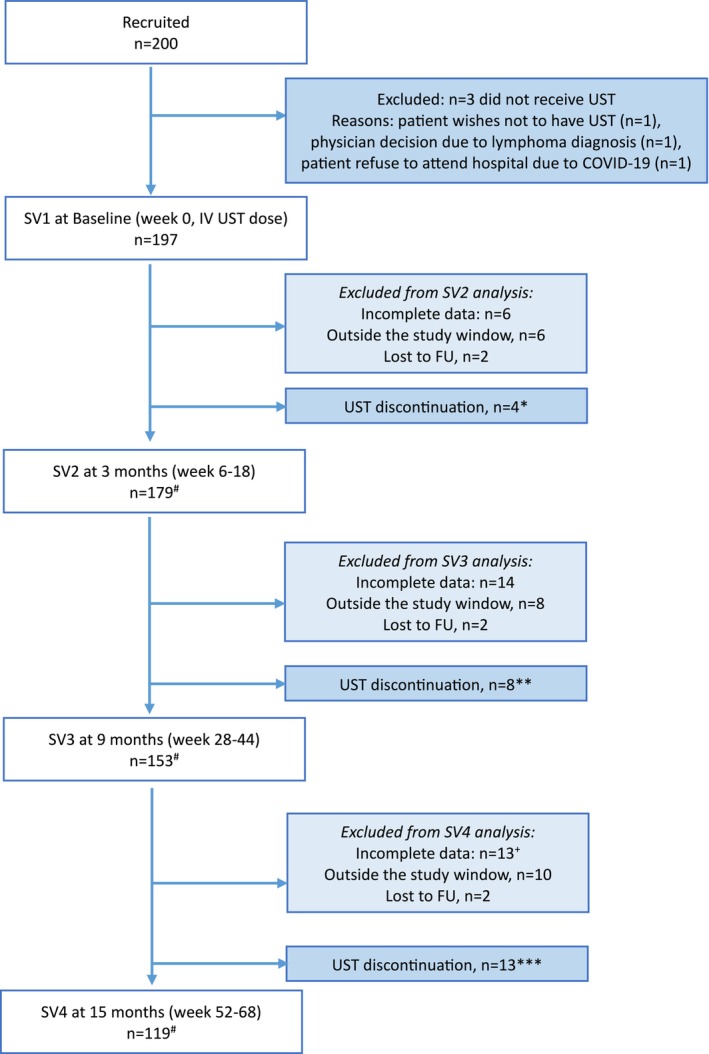
Flowchart of the present study. SV, Study visit. UST, Ustekinumab. FU, follow‐up. ^#^Patients included in the final analysis; *3 patient decision, 1 lack of efficacy; **1 patient decision, 1 side effects, 6 lack of efficacy; ***1 patient decision, and 12 lack of efficacy.

Among the 200 initially recruited patients, data from 197 patients who received UST were analyzed for this study (Figure 1). Included for analysis were patients with complete data and SV within the study windows at each timepoint. Table 1 provides a summary of the clinical and demographic characteristics of the overall population. Patients had a median age of 41 years, 45.2% were male, the median duration of disease was 90 months, and 12.8% were active smokers. The most common CD location was ileocolonic (48.2%), with the majority having non‐stricturing/non‐penetrating disease (57.3%). A history of perianal CD was reported in 9.7% of patients, 30.6% had undergone previous intestinal resection, and 48.2% had a history of EIMs. Among patients with a history of EIMs, 82.1% reported arthralgia, 56.8% had skin or mouth lesions, and 14.7% experienced iritis or uveitis. At baseline, the median PRO2 total score was 14.5 (IQR 6.8–21.0) consistent with moderate disease activity [12]. Most patients (61%; 120 of 197) had elevated biomarkers (CRP and/or FC) at baseline. Specifically, the median CRP was 4.0 mg/L (IQR 1.0–11.0) and the median FC was 399 μg/g (IQR 109–924) at baseline.

**TABLE 1:** Demographic and clinical characteristics of the study population.

Characteristics	*n* = 197
Male, *n* (%)	89 (45.2%)
Age, median yrs. (Q1‐Q3)	41 (29–53)
Disease duration, median mths (Q1‐Q3)	90 (28–182)
BMI, median kg/m^2^ (Q1‐Q3)	25.6 (22.9, 29.4)
Smoking status, *n* (%)	
Current	25 (12.8%)
Former	76 (39.0%)
Never	94 (48.2%)
Disease location (Montreal), *n* (%)	
L1 – Ileal	72 (37.3%)
L2 – Colonic	24 (12.4%)
L3 – Ileocolonic	93 (48.2%)
L4 – Upper GI	4 (2.1%)
Behavior (Montreal), *n* (%)	
B1 – Non‐stricturing, non‐penetrating	110 (57.3%)
B2 – Stricturing	72 (37.5%)
B3 – Penetrating	10 (5.2%)
P – Perianal disease	19 (9.7%)
Prior extra‐intestinal manifestation, *n* (%)	95 (48.2%)
Arthralgia	78 (82.1%)
Iritis/Uveitis	14 (14.7%)
Skin/Mouth lesions	54 (56.8%)
Previous intestinal resection, *n* (%)	60 (30.6%)
Previous use of biologics, *n* (%)	81 (41.1%)
Number of biologics, *n* (%)	
0	116 (58.9%)
1	53 (26.9%)
2	25 (12.7%)
3	3 (1.5%)
Previous exposure of biologics, *n* (%)	
Infliximab	41 (50.6%)
Adalimumab	50 (61.7%)
Vedolizumab	9 (11.1%)
Others^a^	5 (6.2%)
Previous conventional therapy, *n* (%)	
5‐ASA	77 (39.1%)
Immunomodulators	184 (93.9%)
Corticosteroids	183 (92.9%)
Concomitant medications, *n* (%)	
Immunomodulator	98 (50.0%)
6MP	39 (39.8%)
AZA	31 (31.6%)
MTX	20 (20.4%)
6TG	8 (8.2%)
Corticosteroids	70 (36.3%)

^a^ 1 Ustekinumab, 1 Etrolizumab, 1 Ozanimod, 1 anti‐MAP+FMT, 1 unknown investigational product.

Ustekinumab was initiated as the first‐line biologic therapy in 116 (58.9%) patients, while 81 (41.1%) patients had previous exposure to biologics. Among bio‐exposed patients, 65.4% (53 of 81) had failed one biologic therapy, 30.9% (25 of 81) had failed two, and 3.7% (3 of 81) had failed three biologics prior to UST therapy. Among bio‐exposed patients, 50.6% (41 of 81) had previously received infliximab, 61.7% (50 of 81) had received adalimumab, and 11.1% (9 of 81) had been treated with vedolizumab.

At baseline, UST was administered in combination with corticosteroids in 35.3% (70 of 193) of patients, and with immunomodulators, predominantly thiopurines, in 50% (98 of 196) of patients. A combination of both corticosteroids and immunomodulators was prescribed for 35 patients. Among the 70 patients initially on corticosteroids, 52 (74.3%) ceased their usage by the end of the induction phase. Only 5 patients across the entire cohort required the initiation or re‐initiation of corticosteroid therapy subsequently. Regarding patients on concomitant immunomodulators at baseline, 43 of 98 (43.9%) discontinued during the 15‐month follow‐up period. Specifically, 14 patients discontinued between SV1 and SV2, 16 patients discontinued between SV2 and SV3, and the remaining13 patients discontinued between SV3 and SV4.

### 3.2 Clinical Response and Remission

**FIGURE 2:**
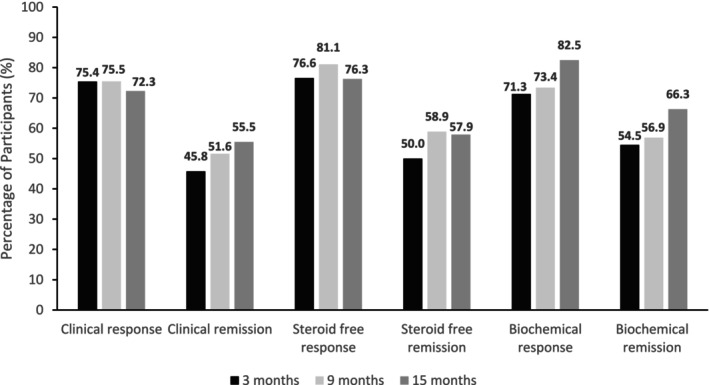
Clinical outcomes at 3, 9 and 15 months. Clinical response was defined as decrease of at least 50% in PRO2 and clinical remission was defined as PRO2 score where abdominal pain ≤ 1 and stool frequency ≤ 3. Corticosteroid‐free response and remission was defined as per definitions of clinical response and remission, and corticosteroid withdrawal in patients who were receiving corticosteroids at baseline. Biochemical response was defined as CRP or FC reduction of at least 50% and biochemical remission was defined as CRP or FC level normalization (5 ≤ mg/L and < 250 μg/g, respectively).

**FIGURE 3:**
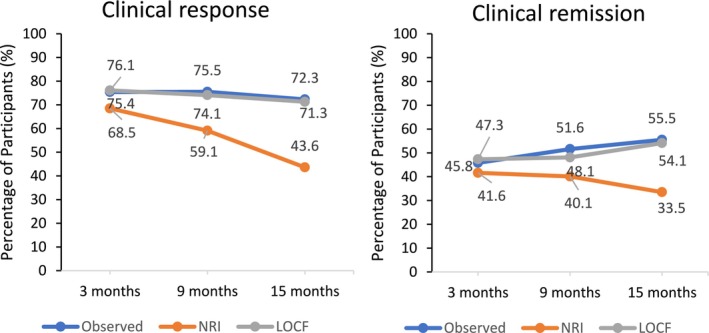
(A) Clinical response and (B) clinical remission at 3, 9 and 15 months, reported using hybrid approaches, including as‐observed (blue line), non‐responder imputation (NRI in orange line), and last observation carried forward (LOCF in gray line) analysis.

Figure 2 illustrates the proportion of patients achieving clinical response and remission at 3, 9 and 15 months during the follow‐up period. Figure 3 presents data on clinical response and remission using a hybrid approach, combining as observed, NRI and LOCF analyses. In the as observed analysis, clinical response rates were 75.4%, 75.5% and 72.3% at 3, 9 and 15 months, respectively. According to NRI analysis, 68.5%, 59.1% and 43.6% of patients achieved clinical response at these time points, while LOCF analysis reported rates of 76.1%, 74.1% and 71.3%. Similarly, clinical remission rates, as observed were 45.8%, 51.6% and 55.5% at 3, 9 and 15 months, respectively. According to NRI analysis, 41.6%, 40.1% and 33.5% of patients achieved clinical remission at these timepoints, while LOCF analysis reported rates of 47.3%, 48.1% and 54.1%.

**FIGURE 4:**
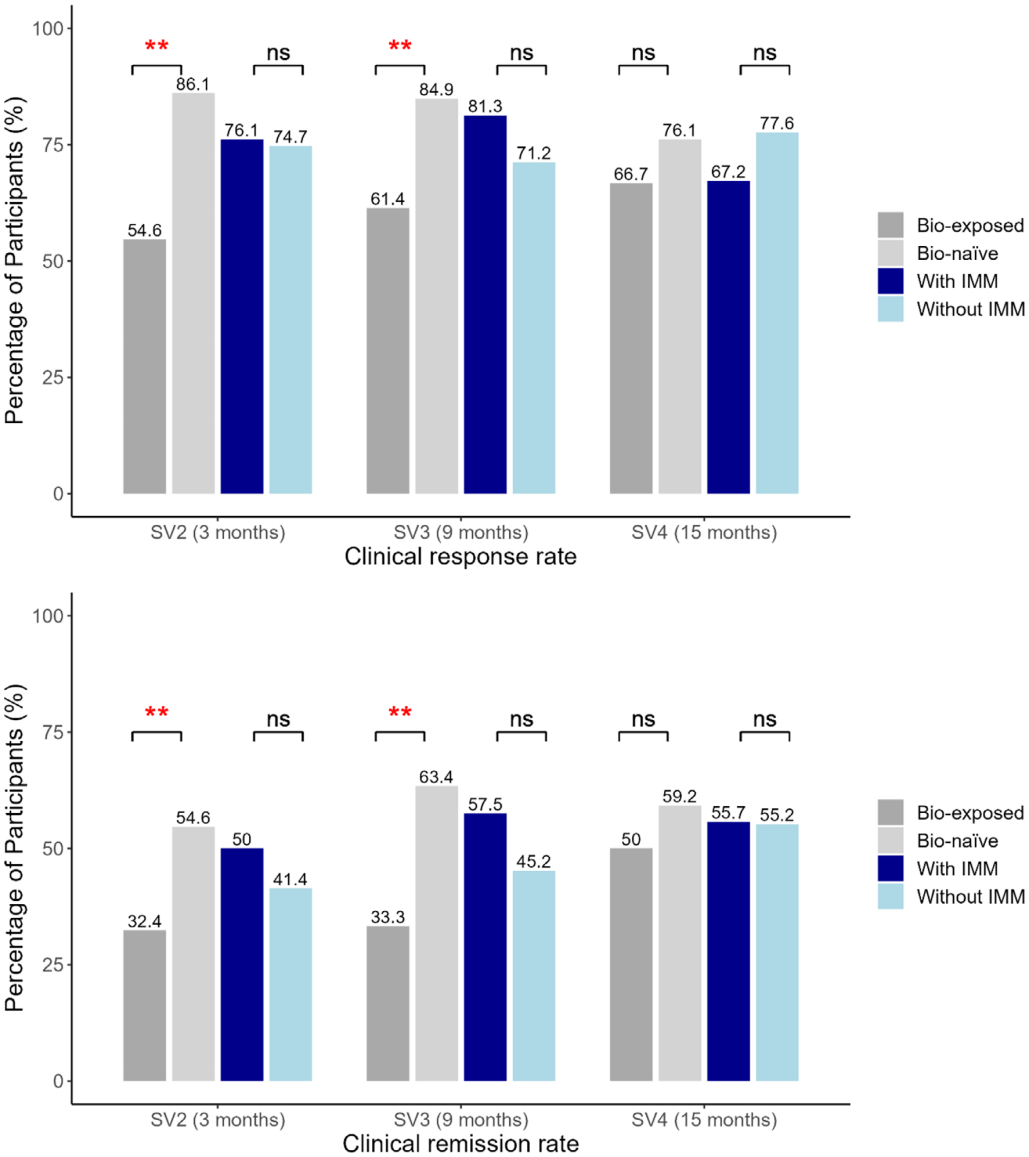
(A) Clinical response and (B) clinical remission at 3, 9 and 15 months stratified by the patient biologic exposure status (blue) and concomitant immunomodulator status (gray). **P‐value < 0.01, *P‐value < 0.05, ns, Non‐significant.

At 3 months post‐induction, clinical response was achieved in 75.4% (135 of 179) of patients, with remission in 45.8% (82 of 179). Notably, higher rates of response (86.1% vs. 54.6%, *p* < 0.01) and remission (54.2% vs. 31.9%, *p* < 0.01) were observed in bio‐naïve patients compared with biologic‐exposed patients (Figure 4). At 9 and 15 months, clinical response rates were 75.5% and 72.3% and clinical remission rates were 51.6% and 55.5%, respectively. Bio‐naïve patients continued to show higher rates of response at 9 months (84.9% vs. 61.4%, *p* < 0.01) and remission (62.2% vs. 31.6%, *p* < 0.01). However, at the 15‐month follow‐up, the differences were not statistically significant (Figure 4). Concomitant immunomodulator use achieved numerically higher rates of clinical response and remission at 3 and 9 months compared with UST monotherapy but there were no significant differences (Figure 4).

Table 2 presents both univariable analysis and multivariable logistic regression models, aiming to identify baseline predictors of clinical response and remission at 3 and 15 months. In the univariable analyses, achieving clinical response at 3 months was inversely associated with disease duration, history of perianal disease, resection, or EIMs, and the number of biologics exposed. Patients with elevated baseline CRP levels were more likely to achieve clinical response. The final predictors in the multivariable model for clinical response at 3 months included a history of EIMs (OR 0.46; 95% CI 0.21–1.00, *p* = 0.05), the number of prior biologic exposures (OR 0.45, 95% CI 0.27–0.72, *p* < 0.001), and elevated baseline CRP levels (OR 3.27; 95% CI 1.39–8.38, *p* = 0.01). Clinical remission at 3 months was inversely associated with female gender, current smoking status, history of resection, EIMs, and the number of biologics exposed at baseline in the univariable analyses. The final predictors in the multivariable model were a history of EIMs (OR 0.43; 95% CI 0.23–0.82, *p* = 0.01) and the number of prior biologic exposures (OR 0.51, 95% CI 0.31–0.80, *p* = 0.004) for clinical remission at 3 months.

**TABLE 2:** Univariable and multivariable predictors of clinical response and remission at 3 and 15 months.

Characteristic	Univariable			Multivariable^a^		
	OR	95% CI	*p*‐value	OR	95% CI	*p*‐value
(A) Clinical response at 3 months						
Age	0.99	0.97, 1.01	0.40			
Sex						
Male	—	—				
Female	0.82	0.40, 1.62	0.60			
BMI (kg/m^2^)	1.03	0.97, 1.09	0.40			
Smoking						
Never	—	—				
Previous	1.08	0.52, 2.27	0.80			
Current	0.83	0.30, 2.58	0.70			
Disease duration	1.00	0.99, 1.00	0.001	1.00	0.99, 1.00	0.09
Behavior						
B1	—	—				
B2	0.83	0.41, 1.71	0.60			
B3	0.60	0.15, 3.00	0.50			
Location						
L1						
L2	0.46	0.16, 1.43	0.20			
L3	0.61	0.27, 1.29	0.20			
L4	0.70	0.08, 14.7	0.80			
Perianal disease^#^	0.42	0.15, 1.24	0.10			
Fistula	0.98	0.22, 6.84	> 0.90			
Resection^#^	0.31	0.15, 0.63	0.001			
EIMs	0.39	0.19, 0.78	0.01	0.46	0.21, 1.00	0.05
Arthralgia	0.50	0.25, 1.00	0.05			
Iritis uveitis	0.71	0.22, 2.75	0.60			
Skin mouth lesions	0.59	0.29, 1.23	0.20			
Biologics number	0.43	0.27, 0.65	< 0.001	0.45	0.27, 0.72	< 0.001
Concurrent Steroid	1.14	0.56, 2.38	0.70			
Concurrent IMM	1.08	0.54, 2.13	0.80			
Elevated CRP	3.02	1.40, 7.13	0.01	3.27	1.39, 8.38	0.01
Elevated FC	0.82	0.41, 1.62	0.60			

(B) Clinical remission at 3 months						
Age	1.00	0.98, 1.02	0.70			
Sex						
Male	—	—				
Female	0.61	0.34, 1.11	0.11			
BMI (kg/m^2^)	0.97	0.91, 1.02	0.20			
Smoking						
Never	—	—				
Previous	0.89	0.48, 1.68	0.70			
Current	0.40	0.13, 1.09	0.08	0.44	0.14, 1.28	0.15
Disease duration	1.00	1.00, 1.00	0.20			
Behavior						
B1	—	—				
B2	0.94	0.51, 1.75	0.90			
B3	0.56	0.11, 2.26	0.40			
Location						
L1	—	—				
L2	1.45	0.55, 3.99	0.50			
L3	0.72	0.38, 1.37	0.30			
L4	1.09	0.13, 9.51	> 0.9			
Perianal disease	0.46	0.14, 1.30	0.20			
Fistula	2.03	0.48, 10.2	0.30			
Resection	0.38	0.19, 0.73	0.005	0.50	0.23, 1.05	0.07
EIMs	0.37	0.20, 0.67	0.001	0.43	0.23, 0.82	0.01
Arthralgia	0.36	0.19, 0.67	0.001			
Iritis uveitis	0.50	0.13, 1.61	0.30			
Skin mouth lesions	0.57	0.29, 1.11	0.10			
Prior IMM	0.45	0.12, 1.56	0.20			
Biologics number	0.50	0.32, 0.76	0.002	0.51	0.31, 0.80	0.004
Concurrent steroid	1.29	0.69, 2.39	0.40			
Concurrent IMM	1.42	0.79, 2.57	0.20			
Elevated CRP	1.39	0.77, 2.54	0.30			
Elevated FC	0.87	0.48, 1.57	0.60			

(C) Clinical response at 15 months						
Age	1.01	0.98, 1.04	0.60			
Sex						
Male	—	—				
Female	1.59	0.71, 3.60	0.30			
BMI (kg/m^2^)	0.97	0.91, 1.04	0.40			
Smoking						
Never	—	—				
Previous	1.07	0.45, 2.57	0.90			
Current	0.77	0.23, 2.81	0.70			
Disease duration	1.00	0.99, 1.00	0.20			
Behavior						
B1	—	—				
B2/B3	1.12	0.50, 2.53	0.8			
Location						
L1	—	—				
L2	1.72	0.45, 8.48	0.50			
L3/L4	1.66	0.71, 3.93	0.20			
Perianal disease	0.64	0.18, 2.60	0.50			
Resection	0.67	0.29, 1.56	0.30			
EIMs	0.45	0.19, 1.02	0.06	0.49	0.20, 1.16	0.11
Arthralgia	0.53	0.23, 1.20	0.13			
Iritis uveitis	0.76	0.14, 5.65	0.80			
Skin mouth lesions	0.64	0.27, 1.53	0.30			
Biologics number	0.70	0.43, 1.13	0.14	0.68	0.41, 1.15	0.15
Concurrent steroid	1.32	0.55, 3.35	0.50			
Concurrent IMM	0.59	0.26, 1.33	0.20			
Elevated CRP	3.24	1.28, 9.41	0.02	2.96	1.07, 9.23	0.05
Elevated FC	2.21	0.98, 5.17	0.06	1.76	0.71, 4.46	0.20

(D) Clinical remission at 15 months^a^						
Age	0.99	0.96, 1.01	0.30			
Sex						
Male	—	—				
Female	0.62	0.29, 1.28	0.20			
BMI (kg/m^2^)	0.94	0.88, 1.01	0.08	0.94	0.88, 1.01	0.09
Smoking						
Never	—	—				
Previous	1.07	0.49, 2.34	0.90			
Current	0.70	0.22, 2.22	0.50			
Disease duration	1.00	0.99, 1.00	0.20			
Behavior						
B1	—	—				
B2/B3	1.02	0.49, 2.12	> 0.9			
Location						
L1	—	—				
L2	2.56	0.72, 10.5	0.20			
L3/L4	1.66	0.77, 3.62	0.20			
Perianal disease	0.64	0.18, 2.26	0.50			
Fistula	3.35	0.48, 66.7	0.30			
Resection	0.67	0.31, 1.44	0.30			
EIMs	0.34	0.16, 0.71	0.005	0.33	0.15, 0.71	0.005
Arthralgia	0.39	0.18, 0.82	0.01			
Iritis uveitis	0.79	0.14, 4.45	0.80			
Skin mouth lesions	0.49	0.22, 1.07	0.08			
Prior IMM	1.26	0.22, 7.06	0.80			
Biologics number	0.76	0.48, 1.19	0.20			
Concurrent steroid	1.09	0.50, 2.42	0.80			
Concurrent IMM	1.02	0.50, 2.11	> 0.90			
Elevated CRP	2.05	0.95, 4.57	0.07	2.05	0.86, 5.07	0.11
Elevated FC	1.91	0.92, 4.02	0.08	1.44	0.63, 3.27	0.40

Abbreviations: CI, Confidence Interval; OR, Odds Ratio.

^a^ All variables with *p* value < 0.2 in the univariable models were included in the multivariable model. Backward regression was used to eliminate variables with *p* values > 0.5.

In the univariable analyses, achieving clinical response at 15 months was inversely associated with a history of EIMs and the number of biologics exposed, while individuals with elevated baseline CRP or FC levels were more likely to achieve clinical response. The final predictors in the multivariable model were elevated baseline CRP levels (OR 2.96, 95% CI 1.07–9.23, *p* = 0.05) for clinical response at 15 months. Clinical remission at 15 months was inversely associated with high BMI and a history of EIMs, while individuals with elevated baseline CRP or FC levels were more likely to achieve clinical remission. The final significant predictor in the multivariable model was a history of EIMs (OR 0.33, 95% CI 0.15–0.71, *p* = 0.005) for clinical remission at 15 months.

### 3.3 Corticosteroid‐Free Clinical Response and Remission

At baseline UST was given concomitantly with corticosteroids in 70 (35.3%) patients and of those 70 patients clinical response rates were 76.6%, 81.1% and 76.3% at 3, 9 and 15 months, respectively (Figure 2). Corticosteroid‐free remission rates were 50.0%, 58.9%, and 57.9% at 3, 9 and 15 months, respectively (Figure 2).

### 3.4 Biochemical Response and Remission

**FIGURE 5:**
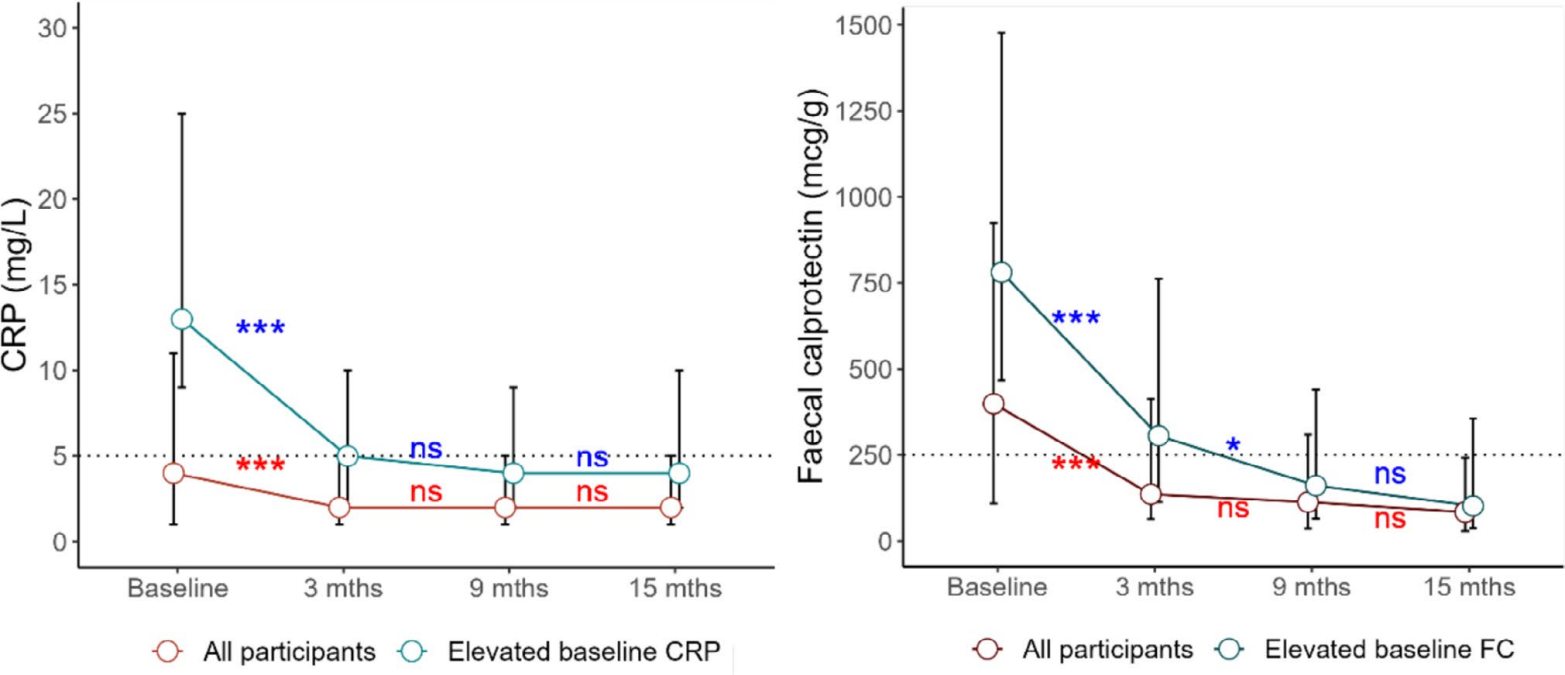
Change in median CRP and FC levels in CD patients treated with UST during follow up in all participants (red line) versus those who had elevated baseline biomarkers (green line). ***P‐value < 0.001, **P‐value < 0.01, *P‐value < 0.05, ns, Non‐significant.

In the overall patient cohort, biochemical response was observed in 71.3%, 73.4% and 82.5%, of patients at 3, 9 and 15 months, respectively (Figure 2). Biochemical remission was achieved in 54.5%, 56.9% and 66.3% at 3, 9 and 15 months, respectively (Figure 2). Among the 120 patients with elevated baseline biomarkers (CRP and/or FC), 74 patients (37.6%) had elevated CRP levels, and 103 patients (52.3%) had elevated FC levels. As illustrated in Figure 5, the trend in biochemical improvement was similar between the overall cohort and in those who had elevated biomarkers at baseline. Specifically, in those with elevated biomarkers at baseline, the median CRP levels significantly decreased from a baseline of 13 mg/L (IQR 9–25) to 5 mg/L at 3 months (IQR 2–10, *p* < 0.001). However, there was no further significant drop observed at 9 months (median CRP 4 mg/L, IQR 2–9, *p* = 0.55) or at 15 months (median CRP 4 mg/L, IQR 2–10, *p* = 0.80). Similarly, the median FC level significantly decreased from a baseline of 781 μg/g (IQR 467–1478) to 306 μg/g at 3 months (IQR 113–762, *p* < 0.001), with a further significant drop at 9 months (161 μg/g, IQR 65–440, *p* = 0.02). However, there was no additional significant drop observed at 15 months (102 μg/g, IQR 37–356, *p* = 0.83).

### 3.5 Impact of Ustekinumab on Extraintestinal Manifestations

**FIGURE 6:**
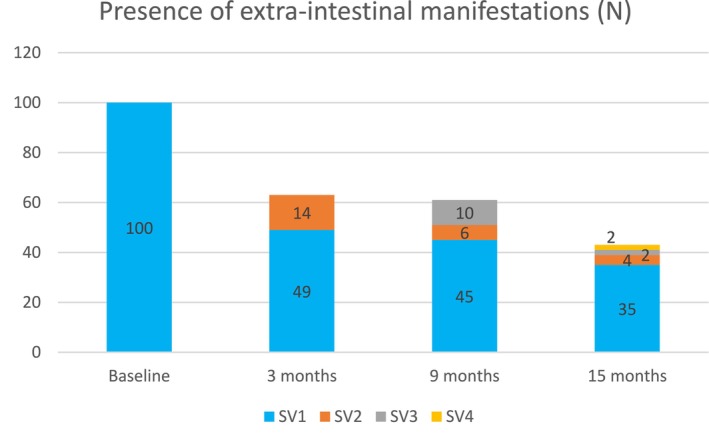
Extra‐intestinal manifestations (EIMs) for participants who reported it at baseline, 3, 9 and 15 months.

All included patients received treatment for active luminal disease, not for EIMs. A prior history of EIMs was reported in 95 (48.2%) patients, and at baseline, 100 patients (50.8%) had active EIMs. This difference, with more patients reporting active EIMs than having a documented history of EIMs was driven primarily by the inclusion of arthralgia. Among those with EIMs at baseline, 51% experienced resolution at 3 months, 55% at 9 months, and 65% at 15 months, as shown in Figure 6.

Among patients with baseline EIMs who experienced resolution, clinical response and remission rates were 80.9%, 75.0%, 80.6% and 42.6%, 58.3%, 52.8% at 3, 9 and 15 months, respectively. During the follow‐up period, 26 patients developed new EIMs, primarily arthralgia. No patients discontinued UST treatment due to these new EIMs. Importantly, the majority of these patients saw resolution of their EIMs over the follow‐up period. Among those who developed new EIMs, a high proportion of patients had achieved either clinical response (79.0%, 70.0%, 50.0% at 3, 9 and 15 months, respectively) or clinical remission (57.1%, 30.0%, 50.0% at 3, 9 and 15 months) respectively.

### 3.6 Dose‐Escalation

All patients received the initial UST dose at approximately 6 mg/kg intravenously. The weight‐based UST doses were 260 mg for 14 patients, 390 mg for 119 patients, and 520 mg for 61 patients (3 cases unknown). For most patients, the second dose was 90 mg SC at week 8. Over the 15‐month follow‐up period, 62 patients (31.5%) received dose escalation at the discretion of the treating clinician. During the induction phase, 14 patients (7.1%) had dose escalation whereby 6 patients received IV re‐induction, and 8 patients received their second dose at week 4 followed by a subsequent dose at week 8. In the maintenance phase, dosing was escalated in 49 patients (24.9%), whereby 37 patients received 90 mg SC 4 weekly, 8 patients received IV re‐induction followed by 90 mg SC 4 weekly, and 4 patients only received IV re‐induction. Among patients who underwent dose escalation during maintenance, 52.2% achieved clinical remission at 15 months.

### 3.7 Persistence

**FIGURE 7:**
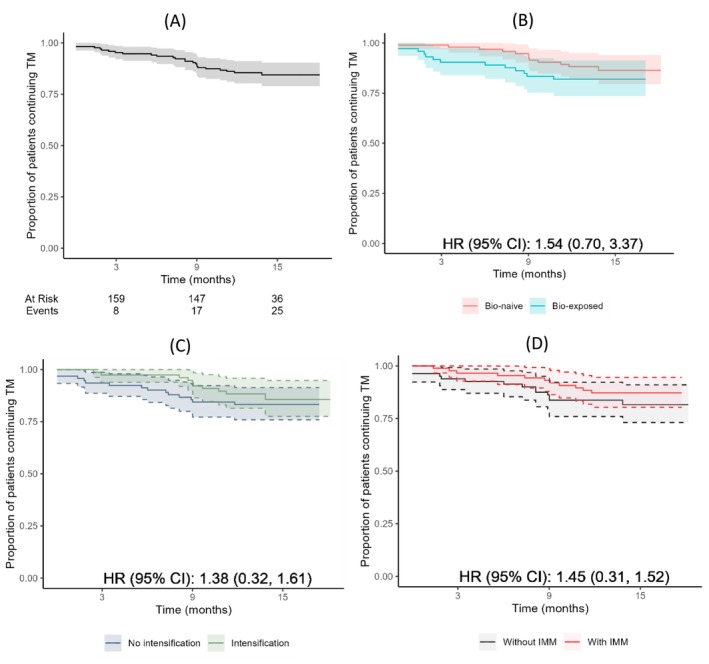
Kaplan–Meier curve showing the failure‐free survival of UST therapy by 15 months of follow‐up. The cumulative probabilities for maintained UST treatment at 3 months, 9 months and 15 months were 95.0% [95% CI 91.3–97.8], 90.0% [95% CI 84.6–94.0], and 84.4% [95% CI 79.3–89.6], respectively. (A) for the whole cohort of patients, (B) bio‐exposure status, (C) dose‐intensification status, and (D) concomitant immunomodulator use.

Among the 197 patients who received IV UST at baseline, 25 patients (12.7%) discontinued treatment over a median period of 13.4 months (IQR 13.1–14.6) due to lack of efficacy in 19 patients, side effects in one patient, and patient preference in 5 patients. Treatment persistence is illustrated in Figure 7. The probability of continuing UST was 95.0% (95% CI 91.3–97.8) at 3 months, 90.0% (95% CI 84.6–94.0) at 9 months, and 84.4% (95% CI 79.3–89.6) at 15 months. Treatment persistence was not affected by prior biologic exposure (HR 1.54; 95% CI 0.70, 3.37), dose escalation (HR 1.38; 95% CI 0.32, 1.61), or concomitant immunomodulator use (HR 1.13; 95% CI 0.34, 2.28).

Among the 119 patients with complete SV4 data, 64 (53.8%) patients maintained a durable clinical response, and 35 (29.4%) patients maintained clinical remission. Patients receiving escalated UST 90 mg SC every 4 weeks had a lower rate of durable clinical remission compared with those on standard 8‐weekly dosing (10.8% (4 of 37) vs. 39.7% (31 of 78); *p* = 0.004).

### 3.8 Post Induction UST Drug Concentration and Its Association With Clinical Outcomes

Ustekinumab serum concentration was measured post‐induction at 3 months in 166 patients, with a median UST level of 9.3 μg/mL (IQR 6.2–13.7) and at 15 months in 89 patients with a median UST level of 6.4 μg/mL (IQR 3.9–10.5). Patients with serum levels obtained within the study window (*n* = 154) had a median UST level of 9.1 μg/mL (IQR 6.1–13.3) after standard induction dosing, measured at a median time of 3.3 weeks (IQR 2.1–5.0) from the last SC dose. This level did not significantly differ in the 8 patients who received SC 4 weekly doses during induction (median UST level 2.6 μg/mL (IQR 1.9–3.0), *p* = 0.10). However, at the 15‐month follow‐up, those receiving 4 weekly SC doses had significantly higher median UST levels (26 of 89, median 10.8 μg/mL, IQR 7.2–13.6) compared with those on standard 8 weekly SC doses (59 of 89, median 5.5 μg/mL, IQR 3.2–8.2, *p* = 0.001).

A strong inverse correlation was observed between CRP and FC levels at 3 months and UST serum concentration levels at 3 months (rho spearman = −0.21, *p* = 0.007 and rho spearman = −0.18, *p* = 0.02, respectively). UST serum concentration at 3 months was significantly associated with biochemical response and remission at 3 months (*p* < 0.001 and *p* = 0.005, respectively) and at 9 months (*p* = 0.03 and *p* = 0.001, respectively). However, no significant association was found between UST serum concentration levels at 3 months and clinical response and remission at 3, 9 and 15 months.

### 3.9 Safety

In total, 161 adverse events were reported, of which 41 AEs led to hospitalization as shown in Table 3. Crohn's disease‐related AEs were reported in 32 patients, infections in 42 patients (predominantly respiratory infections reported in 23 patients, with 11 patients contracting COVID‐19), malignancies in 2 patients, and a UST infusion reaction in 1 patient. These AEs led to a temporary interruption of treatment in 14 patients (8.7%) and discontinuation of treatment in 8 patients (4.0%).

**TABLE 3:** Adverse events reported during 15 months of follow‐up.

Adverse event	Patients (*n*)	Action taken with UST (*n* of patients)
Crohn's disease‐related		
Abscess (perianal)^a^	2	
Flare of disease^a^	19	Drug withdrawn (5), drug interrupted (1), dose increased (4)
New fistula (rectovaginal)	1	Dose increased
Perianal disease	1	
Resection surgery^a^	5	Drug interrupted (4)
Small bowel obstruction^a^	4	Drug withdrawn (1), drug interrupted (1)
Infections		
Candidiasis	1	
Gastrointestinal (appendicitis 1, *Clostridium* ^a^ 1, *Helicobacter* 1, unknown^a^ 4)	7	
Herpes zoster	2	Drug interrupted (1)
Mononucleosis	1	
Oral (dental 2, tonsilitis^a^ 2, unknown 1)	5	Drug interrupted (1)
Respiratory (bronchitis 1, COVID‐19^a^ 11, pneumonia^a^ 2, sinusitis 1, unknown 8)	23	
Urinary (*Proteus* 1, unknown 1)	2	
Uterine	1	
Malignancies		
Melanoma	1	
SCC	1	
Other		
Abdominal pain^a^	13	Drug withdrawn (1)
Abscess (axilla)^a^	1	Drug interrupted
Allergic reaction (not UST‐related)	2	
Alopecia	1	
Anaemia^a^	2	
Arthralgia^a^	5	Drug interrupted (1)
Atelectasis^a^	1	
Cardiac (atrial fibrillation 1, coronary artery disease^a^ 1, carotid artery aneurysm 1, cerebrovascular accident^a^ 1, palpitations 1)	5	Drug interrupted (1)
Choledocholithiasis^a^	1	Drug interrupted
Constipation	1	
Costochondritis	1	
Cytopaenia	1	
Dental caries	1	
Deranged LFTs	2	
Dislocation	1	
Endometriosis^a^	1	
Eye condition	2	
Fatigue	4	
Food bolus	1	
Fracture	4	
Gastric ulcer^a^	1	
Gastrointestinal bleeding^a^	4	Drug interrupted (1)
Headache	5	
Hernia^a^	2	Drug interrupted (1)
Hot flushes	1	
Hypotension^a^	1	
Mental health (worsening of)	3	
Miscarriage	1	
Morton's neuroma	1	
Mouth ulcers	1	
Night sweats	1	
Obesity^a^	1	
Skin condition (benign keratosis 1, other 9)	11	
Tinnitus	1	
UST infusion reaction	1	Drug withdrawn
Vertigo	1	

^a^ Adverse events leading to hospitalization (*n* = 41).

## 4 Discussion

Our study confirms that UST is an effective treatment option for CD, yielding both short‐term and long‐term clinical response and remission rates that are robust. Clinical response rates at 3, 9 and 15 months ranged from 72.3% to 75.5%. Clinical remission rates were also notable, with observed rates ranging from 45.8% to 55.5%. These results align with the existing real‐world evidence of UST in CD management, which has reported early response (3 months) rates of 46.0% to 73.9% and remission rates of 16.0% to 55.6% [13, 14, 15, 16, 17, 18, 19, 20, 21, 22, 23, 24, 25, 26, 27, 28, 29]. Long‐term remission rates (≥ 12 months) from other real‐world studies have varied between 14.0% and 49.0% reflecting some degree of variability between studies, together with variability in a range of other methodological features and patient characteristics [17, 26, 30]. A recent systematic review and meta‐analysis [26] which included 63 studies reported a pooled clinical response rate of 60% (95% CI, 54%–67%) in the short term (8–14 weeks), 64% (57%–71%) in the medium term (16–24 weeks), and 64% (52%–74%) in the long term (48–52 weeks). Remission rates were 37% (28%–46%) in the short term, 42% (36%–49%) in the medium term, and 45% (37%–53%) in the long term. Our study showed higher clinical response and remission rates, likely attributable to the high proportion of bio‐naïve patients and patients without biochemical activity at baseline (39%) in our cohort. Variations in the definition of clinical outcomes used in prior studies compared with ours which adopted definitions from the STRIDE II consensus statement may also contribute to these differences.

Stratifying by prior biologic exposure status demonstrated consistently higher clinical response and remission rates in bio‐naïve patients compared with bio‐exposed patients, particularly at 3 and 9 months, although the differences diminished by 15 months. A similar trend was observed in a single centre Canadian retrospective study by Sedano et al. [31], which is the only other study powered to examine the effect of prior biologic exposure. These findings collectively emphasize the potential benefits of using UST as a first‐line biologic therapy.

Our analysis identified several predictors of clinical response and remission. The presence of EIMs, a history of intestinal resection, and a history of multiple biologic failures, were associated with a lower probability of achieving clinical response and remission. On the other hand, an elevated baseline CRP level was associated with a higher likelihood of clinical response and remission. These findings align with previous studies, which have identified similar negative and positive predictive factors [15, 18, 32, 33]. Other predictive factors identified in previous studies that were not observed in our study include raised body mass index [32], which was associated with lower rates of steroid‐free remission, younger age at diagnosis and current smoking, which were associated with poor response and remission [21, 27], and stricturing phenotype, which was associated with similarly poor outcomes [17]. Our failure to replicate these findings may relate to the use of dose‐escalation in patients at the discretion of the treating clinicians and the relatively low rates of smoking in our cohort.

Regarding safety, this real‐world study reported a higher number of AEs compared with previous studies, with 161 cases of AEs in 197 patients, 41 of which required hospitalization. The COVID‐19 pandemic likely contributed to these findings, as respiratory infections were the most frequently reported AEs, including 11 cases of confirmed COVID‐19 infection. Importantly, despite the high number of reported AEs, they led to temporary treatment interruption in only 8.7% of cases and treatment discontinuation in only 4.0%, suggesting that AEs were mainly attributable to active CD requiring dose escalation or were mild in nature, not directly related to the drug itself, and did not require treatment discontinuation. In comparison, real‐world studies reported AE incidence rates of 26% (95% CI, 25–27) and discontinuation rates of 6.8% [26]. While our reported AE incidence rates were higher than those in other real‐world studies, the discontinuation rate was lower, and may reflect comprehensive capture of AEs in this prospective study. Comparatively, systematic reviews and meta‐analyses have shown no significant differences in AE rates between UST and placebo groups in clinical trials, or when comparing high and low UST doses [34, 35].

Our study has several notable strengths. Our approach included prospective follow‐up with a substantial cohort size and nationwide coverage, which enabled us to construct a representative cohort reflective of routine clinical care and distinct from previously reported real‐world cohorts. We recruited a relatively homogenous patient population characterized by moderate to severe CD with a high prevalence of concomitant immunomodulator use. Moreover, our study included a substantial number of patients where UST was employed as a first‐line biologic therapy. Our cohort consisted of approximately equal proportions of bio‐naïve and bio‐exposed patients, along with a comparable distribution of patients receiving concomitant immunomodulators and those on UST monotherapy. This balanced representation allowed for meaningful comparisons between several patient subgroups, and offered insights that may address current gaps in knowledge.

Limitations of this study include instances where study visits fell outside the designated study window or contained incomplete or missing data, attributable to various patient‐ or site‐related factors or patients lost to follow‐up. The lack of reliable endoscopic or imaging (MRI or intestinal ultrasound) data, and focus on clinical and biochemical data may be seen as a further limitation. Additionally, the study faced considerable challenges posed by the COVID‐19 pandemic during the follow‐up period, affecting in‐person study visits and data collection including blood and stool tests. To address the challenges of COVID‐19 restrictions on in‐person visits patients were encouraged to engage in telehealth consultations and remote sample and biospecimen collection, ensuring their continued clinical care and participation in the study.

## 5 Conclusions

In conclusion, our real‐world prospective study on the efficacy and safety of UST in managing moderate to severe CD in Australia offers valuable insights. With a large cohort with 15 months of follow‐up data consisting of a substantial proportion of bio‐naïve patients and those receiving concomitant immunomodulators, our findings confirm UST as an effective treatment option for CD, with consistently high clinical response and remission rates in short‐term and long‐term follow‐up. Notably, bio‐naïve patients have significantly higher response rates in the short term as compared to bio‐exposed patients; however bio‐exposed patients achieve comparable response and remission rates by 15 months of treatment. The impact of concomitant immunomodulator use remains a subject of interest, with no significant effect seen in our cohort. Safety concerns were largely manageable. Our study improves our understanding of UST therapy and informs its role as a first‐line treatment option for CD patients.

## Funding

This work was supported by Janssen‐Cilag Pty Ltd. through clinical investigator‐initiated study research support grant (Grant number: CNTO1275RD4024). The funding body had no role in the design of this study and will not have any role during its execution, analyses, interpretation of the data or decision to submit results.

## Ethics Statement

Ethics approval for this research project has been granted for all study sites by the Royal Brisbane & Women's Hospital Human Research Ethics Committee (HREC/2019/QRBW/49250) and the Mater Misericordiae Ltd. Human Research Ethics Committee (HREC/MML/56171). The study protocol has been registered on the Australian New Zealand Clinical Trials Registry (ACTRN12623000844640).

## Conflicts of Interest

Y.‐K.A. reports grants from Janssen, during the conduct of the study; has received speaking and consulting fees from Abbvie, Bristol Myers Squibb, Celltrion, Chiesi, Dr. Falk, Ferring, Janssen, Pfizer, Sandoz, Shire and Takeda; served on advisory boards as a member for Abbvie, Bristol Myers Squibb, Chiesi, Janssen, NPS Medicine Wise, and Microba; received research and educational funding from Abbvie, Celltrion, Dr. Falk, Janssen, Pfizer, Sandoz and Takeda. E.K. has received research grants from Pfizer and Celltrion; speaker fees from Ferring. L.T. has received advisory board fees from Abbvie, Janssen, Chiesi, Takeda, Pfizer, BMS, and Celltrion, and research grant support from Pfizer, Abbvie and Celltrion. D.L. has received consultation fees from Eli Lily, and AbbVie, educational funding from BMS, Pfizer and Janssen, research grants from Ferring and Janssen, speaker fees from Dr. Falk. S.J.C. has received honoraria for Advisory Board participation, speaker fees, educational support and/or research support from Abbvie, Amgen, BMS, Celltrion, Chiesi, Dr, Falk, Eli Lilly, Ferring, GSK, Janssen, MSD, Organon, Pfizer, Sandoz, Takeda, Agency for Clinical Innovation, Medical Research Future Fund, South Western Sydney Local Health District, Sydney Partnership for Health, Research and Enterprise (SPHERE) and The Leona M and Harry B Helmsley Charitable Trust. R.B. has received grant/research support/speaker honoraria/advisory board fees from AbbVie, Ferring, Janssen, Shire, Takeda, GlaxoSmithKline, Bristol Myers Squibb, and Emerge Health; and is a shareholder in Biomebank. N.S.D. has received speaking and consulting fees from Janssen, AbbVie, Ferring, Shire, Takeda, Chiesi; served on advisory boards for Janssen, AbbVie, Shire, Takeda, Celltrion and Chiesi; and has received research and educational funding from Abbvie, Janssen, Pfizer, and Takeda. P.D.C. is supported by a NHMRC Emerging Leader 2 Fellowship and has received research support from Janssen, AbbVie, Ferring, Shire, Takeda and Celltrion; served on advisory boards for Janssen, AbbVie, Ferring, Shire, Takeda, Celltrion and Chiesi; and has received speaking and consulting fees from Janssen, AbbVie, Ferring, Shire, Takeda, Celltrion, Pfizer and Baxter. C.F.D.L.W.S. is supported by a National Health and Medical Research Council (NHMRC) postgraduate scholarship and has received research educational support from Pfizer and research funding from the Robert C Bulley Charitable Foundation and the St Vincent's Hospital Melbourne Research Endowment Fund. S.G. has received honoraria for Advisory Board participation, education support and/or research support from Ferring, Abbvie, Janssen, Pfizer, Sandoz, Takeda and Dr. Falk. B.C. has received speaking and consulting fees from Abbvie, Celltrion, Chiesi, Dr. Falk, Ferring, GSK, Janssen, Pfizer, Sandoz, Shire, and Takeda; served on advisory boards as a member for Abbvie, Chiesi, Celltrion, Janssen, GSK; and received research and educational funding from Abbvie, Dr. Falk, Janssen, Pfizer, and Takeda. M.P.S. has received educational grants or research support from Gilead and Celltrion, speaker fees from Janssen, Abbvie, Ferring, Takeda, Pfizer, Shire, Celltrion, Eli Lilly, and Dr. Falk Pharma; and has served on advisory boards or received consultancy fees from Janssen, Takeda, Pfizer, Celgene, Abbvie, MSD, Emerge Health, Gilead, BMS, Celltrion, and Eli Lilly. L.S.W. has received educational support and/or speaker fees from Shire, BMS, Ferring, Sandoz, Janssen, Pfizer Fresenius Kabi, and the Harry B Helmsley Charitable Trust. R.W.L. advisory board membership: AbbVie, Aspen, BMS, Celgene, Celltrion, Chiesi, Ferring, Glutagen, Hospira, Janssen, Lilly, MSD, Novartis, Pfizer, Prometheus Biosciences, Takeda; research grant support: Celltrion, Shire, Janssen, Takeda, Gastroenterological Society of Australia, NHMRC, Gutsy Group, Pfizer, Joanna Tiddy grant from the University of Sydney, McCusker Charitable Foundation. G.R.‐S. has served on advisory boards for Janssen, Novartis, Takeda, Ferring, Abbvie, Organon, GSK, and Pfizer; has received research funding from Janssen; and has received speaker fees from Janssen, Takeda, Pfizer, Ferring, Novartis, and GSK. J.B. has received speaking and consulting fees from Abbvie, Bristol Myers Squibb, Celltrion, Chiesi, Dr. Falk, Ferring, Janssen, Pfizer, Sandoz, Shire, and Takeda; served on advisory boards as a member for Abbvie, Bristol Myers Squibb, Chiesi, Janssen, NPS Medicine Wise, Anatara, Microba; received research and educational funding from Abbvie, Janssen, Pfizer, and Takeda. N.L., N.A., A.A., H.P., K.S. did not have any conflicts of interest.

## Data Availability

The data that support the findings of this study are available on request from the corresponding author. The data are not publicly available due to privacy or ethical restrictions.
